# Endoscopic diagnosis of Whipple disease in a patient without gastrointestinal symptoms: A case report

**DOI:** 10.1002/deo2.37

**Published:** 2021-09-01

**Authors:** Yujiro Henmi, Yuki Hirata, Eiko Koubayashi, Azusa Hara, Yutaka Naka, Kazuki Kakimoto, Sadaharu Nouda, Ken Kawakami, Toshihisa Takeuchi, Kazuhide Higuchi

**Affiliations:** ^1^ Moriguchi Keijinkai Hospital Osaka Japan; ^2^ Second Department of Internal Medicine Osaka Medical and Pharmaceutical University Osaka Japan; ^3^ Midorigaoka Hospital Osaka Japan; ^4^ Katsuragi Hospital Osaka Japan

**Keywords:** electrons, endoscopy, microscopy, real‐time polymerase chain reaction, tropheryma, whipple disease

## Abstract

Whipple's disease is a systemic chronic bacterial infection caused by *Tropheryma whipplei*, a gram‐positive bacillus. *T. whipplei* infection in the small intestine often causes malabsorption and is often accompanied by gastrointestinal symptoms such as diarrhea and abdominal pain. In this report, we describe our experience with a case of Whipple's disease in which the affected patient did not have the typical gastrointestinal symptoms. The patient was an 80‐year‐old male who presented with complaints of weight loss and lower leg edema due to malabsorption and shortness of breath during exertion. A blood test revealed a decreased albumin level and an elevated C‐reactive protein level. Endoscopic images revealed diffuse white villi, the presence of which extended from the duodenum to the upper jejunum. We made a diagnosis of Whipple's disease based on pathological findings associated with the duodenum, electron microscopic findings, and findings of polymerase chain reaction (PCR) tests (performed using mucosal tissue). Clinical symptoms and endoscopic findings improved with antibiotics. Real‐time PCR tests were performed for a quantitative evaluation of the effect of treatment. Endoscopy is useful for diagnosing Whipple's disease when there is an absence of gastrointestinal symptoms, and hypoalbuminemia of unknown etiology is observed.

## INTRODUCTION

Whipple's disease was first reported by George Whipple in 1907,^1^ and to date, approximately 1000 cases have been reported worldwide.^2^ This disease commonly affects middle‐aged Caucasians; to the best of our knowledge, only about 10 cases involving Japanese patients with Whipple's disease have been reported in the literature. With respect to genetic backgrounds, it has been suggested that there is an association between the human leukocyte antigen B27 (HLA‐B27) and Whipple's disease. The prevalence of this disease is believed to be low in Japan due to the low number of HLA‐B27‐positive Japanese people.^3^ It is thought that *Tropheryma whipplei* infection causes this disease; *T. whipplei* cells were observed with an electron microscope in 1961,^4^ and the bacterium was successfully cultivated in 2000.^5^ Herein, we report a case of a patient who could be diagnosed with Whipple's disease through early endoscopic examination, despite not presenting with gastrointestinal symptoms.

### Case report

**FIGURE 1:**
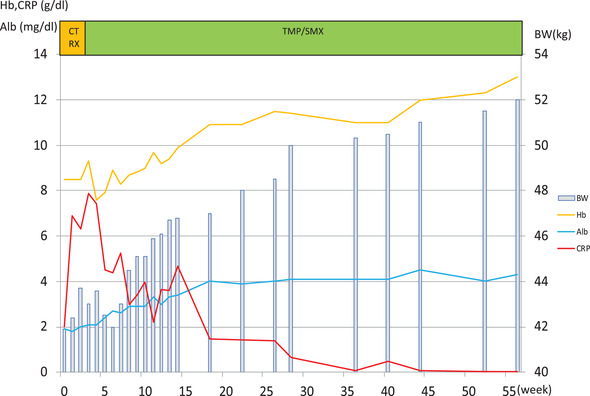
Clinical course of the patient after first admission. Through treatment with antibiotics, the patient's condition improved with respect to the inflammatory markers, and his nutritional status gradually recovered Abbreviations CTRX, ceftriaxone; TMP/SMX, trimethoprim‐sulfamethoxazole.

**FIGURE 2:**
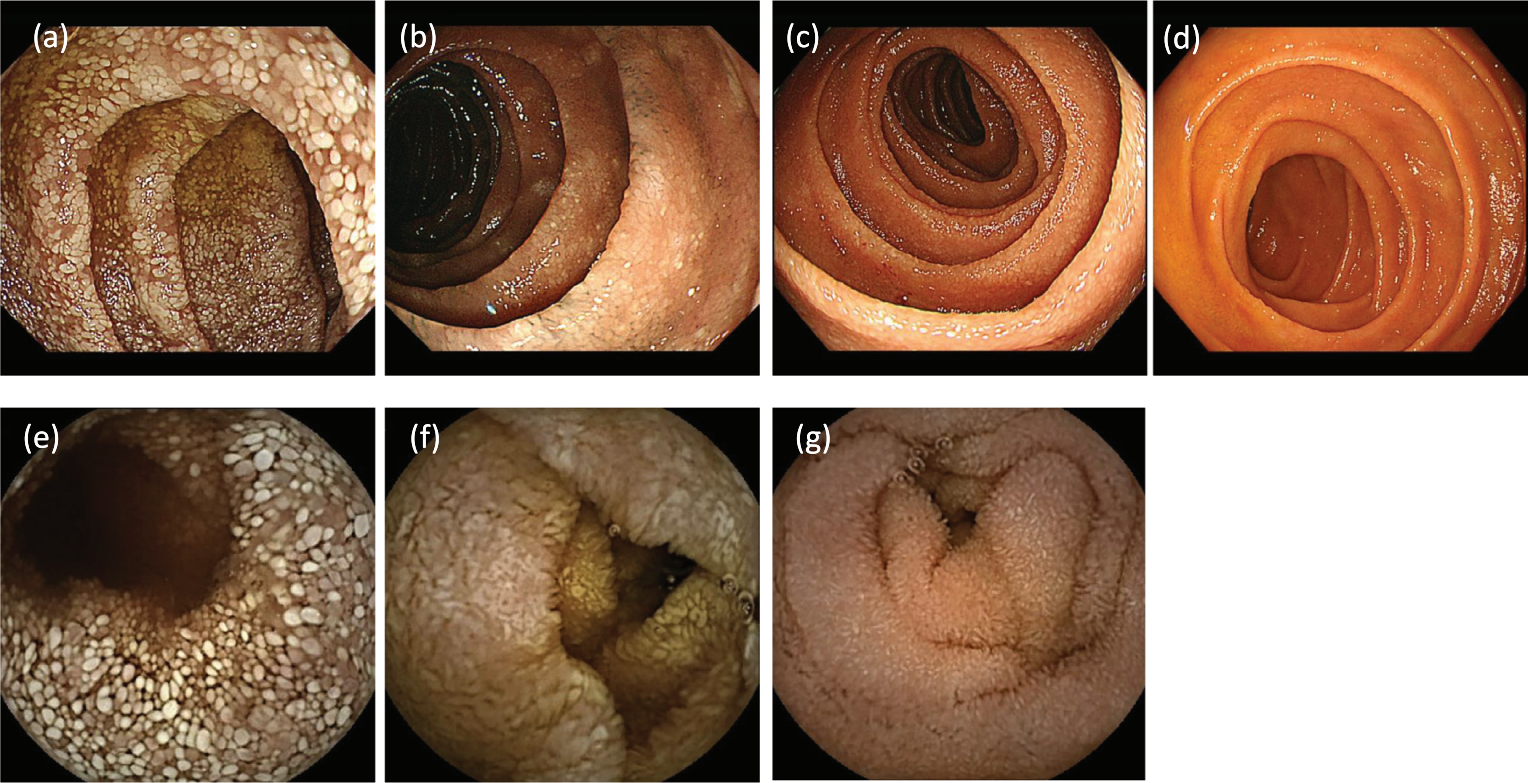
Endoscopic findings associated with the duodenum that were obtained at the time of admission (a), 3 months after the start of treatment (b), 9 months after the start of treatment (c), and 2 years after the start of treatment (d). Capsule endoscopy findings obtained at admission (e), 2 weeks after the start of treatment (f), and 1 year after the start of treatment (g)

**FIGURE 3:**
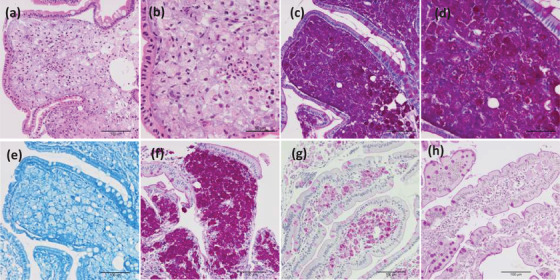
Histopathological findings. (a–e) Histological features of the specimens derived through a duodenum biopsy that was performed at the time of admission; (a and b) Hematoxylin and eosin staining; (c and d) periodic acid‐Schiff (PAS) staining; (e) Ziehl–Neelsen staining. (f–h) Histological features of the duodenum (associated with PAS staining) observed through endoscopic examinations performed during the follow‐up period; (f) 3 months after the start of treatment, (g) 9 months after the start of treatment, (h) 2 years after treatment. Original magnification; (a) × 200, (b) × 400, (c) × 200, (d) × 400, (e) × 200, (f–h) × 200.

An 80‐year‐old male (Japanese, East Asian) visited our hospital and complained of weight loss (from 50 kg to 41.9 kg), which had occurred within 2 months, and shortness of breath during exertion without any digestive symptoms. He was born in Osaka, Japan, and his occupation is office worker; he had never worked with sewage. Physical examination performed at the time of admission revealed the following: height, 160 cm; body weight, 41.9 kg; body mass index, 16.37 kg/m^2^; body temperature, 36.5°C; blood pressure, 116/54 mm Hg; and heart rate, 78 beats/min. A blood test revealed a decreased albumin level and elevated C‐reactive protein level (Figure 1). Anti‐HTLV‐1 antibodies were not detected in the serum. Upper gastrointestinal endoscopy revealed diffuse enlarged white villi in the duodenum (Figure 2a). A histopathological examination revealed the accumulation of foamy macrophages in the submucosa (Figures 3a and 3b). Periodic acid**–**Schiff (PAS) staining revealed the cytoplasmic granules of the macrophages to be strongly PAS positive (Figures 3c and 3d). Furthermore, Ziehl–Neelsen staining did not identify any acid‐fast microorganisms (Figure 3e). The findings of capsule endoscopy were similar to those of upper endoscopy; lesions were found up to about one‐third of the entire small intestine on the mouth side (Figure 2e).

**FIGURE 4:**
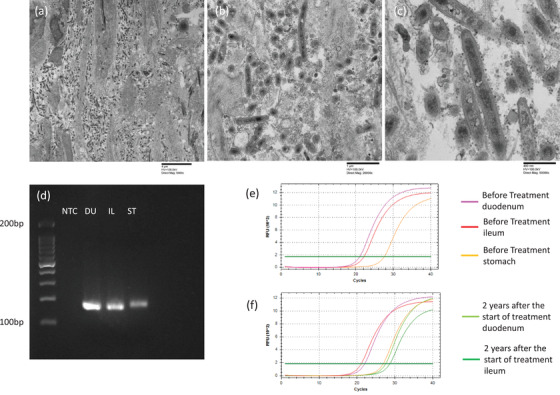
(a–c) Electron microscopic findings through which *Tropheryma whipplei* was detected. Original magnification; (a) × 5000, (b) × 20000, (c) × 50000. (d–f) Detection of *T. whipplei* through a polymerase chain reaction (PCR) test. (d) DNA amplification was observed in the tissue samples from the duodenum and ileum obtained through a biopsy performed at the time of admission. (e) Detection of *T. whipplei* DNA using a real‐time PCR test performed with 16s rDNA‐derived primers using biopsy specimens obtained at the time of admission. (f) A real‐time PCR test using biopsy specimens obtained at the time of 2 years after treatment Abbreviations: DU, duodenum; IL, ileum; NTC, no template control; ST, stomach.

Colonoscopy showed no obvious abnormal findings. Because Whipple's disease was suspected based on the endoscopic and histological findings, electron microscopy and polymerase chain reaction (PCR) examinations using biopsy tissues were used to establish a definitive diagnosis. Electron microscopy revealed the presence of rod‐shaped bacteria with trilamellar cell walls in the submucosa of the duodenal mucosa (Figures 4a–4c). For the detection of *T. whipplei*, a PCR test was performed using tissue obtained from a duodenal biopsy, the result of which was positive (Figure 4d). The results of a PCR test performed after admission showed that the cycle threshold (Ct) value for the duodenal and jejunal samples was around 21–22, whereas the Ct value for the gastric sample was 26. We used primers W3AF and W4AR to target a 159‐base‐pair fragment of the 16s ribosomal RNA of *T. whipplei* (Figure 4e).

After a definitive diagnosis had been formulated, we started the administration of an antibiotic (ceftriaxone, 2 g/day). Two weeks after the start of antibiotic administration, upper endoscopy and capsule endoscopy were performed to determine the effect of the treatment; the findings revealed that although white villi were still present, the patient's condition had improved since the time of admission (Figure 2f). For the provision of maintenance therapy, we started administering a combination of sulfamethoxazole and trimethoprim (1920 mg/day); the patient's hypoalbuminemia gradually improved, and he was discharged from the hospital 14 weeks after the start of treatment. At the time of discharge, the patient's symptoms of shortness of breath were gone, and his weight was over 46 kg. The patient's albumin level normalized 20 weeks after we started the oral administration of the sulfamethoxazole‐trimethoprim combination (Figure 1). Endoscopy was performed 3 months after the start of the treatment, and although the white villi were noted to have persisted (Figure 2b). Unlike the endoscopic findings, there were no significant differences between the findings of the histological examinations performed before treatment and 3 months after the start of treatment (Figure 3f). The findings of endoscopy performed 9 months after the start of treatment were almost normal (Figure 2c), and the findings of a histological examination performed at this time revealed that the number of PAS‐positive macrophages had reduced markedly (Figure 3g). Presently, 2 years have passed since the start of the treatment, and with continued maintenance therapy, no apparent recurrence has been observed endoscopically (Figure 2d). Biopsy tissue examination after 2 years of treatment revealed that a small number of PAS‐positive cells had remained in the submucosa (Figure 3h). Results of a PCR test performed using the tissue specimens from the duodenum or jejunum showed that the *T. whipplei* copy number (Ct value: approximately 27–28) was clearly lower than the number of copies at admission (Figure 4f).

## DISCUSSION

Whipple's disease is a systemic chronic bacterial infection caused by *T. whipplei*, a gram‐positive bacillus. It manifests as a malabsorption syndrome, and the main symptoms associated with this disease are diarrhea, weight loss, abdominal pain, arthralgia, and the presence of small intestinal lesions, including lesions in the duodenum.

Endoscopic findings associated with the diffusely enlarged white villi of the duodenum, which are a characteristic of this disease, have become widely known, and a few cases involving Japanese patients have been reported. Endoscopic differential diseases with white villi in the duodenum include lymphangiectasia, nontuberculous mycobacterial infection, follicular lymphoma, and strongyloidiasis. It is not easy to differentiate by endoscopic findings alone, but it is necessary to differentiate based on histopathological findings.

The presence of many PAS‐positive macrophages in the duodenal submucosa is one of the suspicious findings associated with Whipple's disease. However, this finding is not specific to the disease, and for a definitive diagnosis, PCR tests and electron microscopy are necessary for proving the presence of *T. whipplei*.^6^ The results of the PCR tests must be carefully interpreted due to the possibility of contamination and the presence of carriers among healthy individuals.^7^ In this regard, although there are concerns about false positives in the results of PCR test by electrophoresis method, *T. whipplei* DNA has recently been quantitatively detected using real‐time PCR tests.^8^ We also performed real‐time PCR tests using duodenal, jejunal, and gastric biopsy specimens. In our case, the endoscopic findings of the duodenum showed a tendency to improve after 2 weeks of antibiotic administration, and improved to almost normal findings with only a few white villi remaining after 9 months. A previous report that followed the endoscopic findings for a long period of time also reported that the endoscopic findings normalized about 7 months after the start of treatment.^9^

The findings of endoscopy performed 2 years after the start of the treatment were almost normal in this case; however, with respect to the pathological features, we found that PAS‐positive macrophages remained in the submucosa after treatment. It is believed that after remission, PAS‐positive macrophages remain in the submucosa for a certain length of time, and the effect of treatment should be judged based on the results of the PCR tests.^7^ In this case, the results of the real‐time PCR tests clearly showed that the amount of *T.whipplei* DNA in the duodenum and jejunum tissues was decreased after the start of treatment, indicating that remission was maintained.

The frequency of each symptom of Whipple's disease was reported to be 76% for diarrhea, 55% for abdominal pain, 92% for weight loss, and 91% for hypoalbuminemia.^10^ In other words, symptoms of malabsorption are more common than gastrointestinal symptoms in Whipple's disease. If there is unexplained hypoalbuminemia, even in the absence of gastrointestinal symptoms, upper endoscopy should be performed with consideration of Whipple's disease as a differential.

## CONFLICT OF INTERESTS

Authors declare no conflict of interests for this article.

## ETHICS STATEMENTS

All procedures performed in studies involving human participants were in accordance with the ethical standards of the institutional and/or national research committee and with the 1964 Helsinki declaration and its later amendments or comparable ethical standards. Written informed consents were obtained from from all subjects for publication of this case report.

## FUNDING INFORMATION

None.
